# Incidence of endocrine-related immune-related adverse events in Japanese subjects with various types of cancer

**DOI:** 10.3389/fendo.2023.1079074

**Published:** 2023-01-23

**Authors:** Yuichiro Iwamoto, Tomohiko Kimura, Hideyuki Iwamoto, Junpei Sanada, Yoshiro Fushimi, Yukino Katakura, Fuminori Tatsumi, Masashi Shimoda, Shuhei Nakanishi, Tomoatsu Mune, Kohei Kaku, Hideaki Kaneto

**Affiliations:** Division of Diabetes, Endocrinology and Metabolism, Kawasaki Medical School, Kurashiki, Japan

**Keywords:** immune-related adverse events, immune check point inhibitors, cytotoxic T lymphocyte antigen-4 inhibitors, programmed cell death protein 1 inhibitor, programmed cell death protein 1 ligand 1 inhibitors

## Abstract

**Background:**

Immune checkpoint inhibitors (ICIs), such as cytotoxic T lymphocyte antigen-4 (CTLA-4) inhibitors, programmed cell death protein 1 (PD-1) inhibitors, and programmed cell death protein 1 ligand 1 (PD-L1) inhibitors, are often used to treat a variety of malignancies. ICIs are known to cause endocrine-related immune-related adverse events (irAEs), but the incidence varies among reports and/or agents. This study evaluated the incidence of endocrine-related irAEs in patients who were treated with ICIs in Japan.

**Method:**

This single-center, retrospective, observational study examined the incidence and clinical characteristics of endocrine-related irAEs in 466 participants who were treated with ICIs at Kawasaki Medical School Hospital.

**Result:**

The mean age of participants with and without endocrine-related irAEs was 69.1 ± 1.8 years and 68.1 ± 1.1 years, respectively, with no difference between them. The overall incidence of any endocrine-related irAEs among the participants was 25.5%. Hypothyroidism was prevalent in 24.3%, hypoadrenocorticism in 3.2%, hypopituitarism in 0.9%, and insulin-dependent diabetes mellitus in 1.1%. Participants receiving combination therapy with CTLA-4 and PD-1 inhibitors had a significantly higher incidence of endocrine-related irAEs than those receiving monotherapy.

**Conclusion:**

Endocrine-related irAEs correlated significantly with survival and mean observation period. There was substantial difference in the incidence of endocrine-related irAEs among various types of ICIs and types of cancer. We should bear in mind that endocrine testing is necessary during the treatment with ICIs.

## Introduction

Immune checkpoint inhibitors (ICIs) are one of the therapeutic agents used against a variety of cancer tumors. ICIs which are currently in clinical use include cytotoxic T lymphocyte antigen-4 (CTLA-4) inhibitors, programmed cell death protein 1 (PD-1) inhibitors, and programmed cell death protein 1 ligand 1 (PD-L1) inhibitors.

Immune-related adverse events (irAEs) associated with the treatment of ICIs have become common and can affect endocrine organs. Reported endocrine-related irAEs include destructive thyroiditis, hypoadrenocorticism, hypopituitarism, and insulin-dependent diabetes mellitus. Destructive thyroiditis is commonly observed after monotherapy with PD-1/PD-L1 inhibitors. It is essential to perform prompt therapeutic intervention for severe hypothyroidism, hypoadrenocorticism, and insulin-dependent diabetes mellitus.

It has been reported that the incidence of irAEs with CTLA-4 inhibitors is 0-17% for hypopituitarism, 4.3-11.0% for hypothyroidism, 2.0% for hyperthyroidism, and <2% for hypoadrenocorticism. Similarly, it has been reported that PD-1/PD-L1 inhibitors cause hypopituitarism in less than 1%, hypothyroidism in 5.9%, hyperthyroidism in 1.0-4.7%, insulin-dependent diabetes in 0-1%, and adrenocortical hypofunction in less than 2% (1). On the other hand, there are few reports of incidents of endocrine-related irAEs in Asian and Japanese subjects. This study evaluated the incidence of endocrine-related irAEs in patients who were treated with ICIs in Japan, and the difference in life expectancy between patients with and without irAEs.

## Material and methods

### Study population and patient preparation

This study is a single-center, retrospective observational study. A total of 466 patients who were treated with ICIs at Kawasaki Medical School Hospital from September 1, 2014, to May 31, 2022, were included in this study. The research protocol including opt-out informed consent was approved by the Institutional Review Board of Kawasaki Medical School (No. 5726-00). The study was conducted in accordance with the principles of the Declaration of Helsinki. The ICIs which were used in our hospital are the CTLA-4 inhibitor ipilimumab, the PD-1 inhibitors nivolumab and pembrolizumab, and the PD-L1 inhibitors atezolizumab, durubumab, and avelumab. Twenty-five patients received the combination therapy of ipilimumab and a PD-1 inhibitor, 189 received nivolumab alone, 162 received pembrolizumab alone, 73 received atezolizumab alone, 13 received dulubumab alone, and 4 received avelumab. The incidence of endocrine-related irAEs was investigated based on medical records and laboratory results.

### Diagnosis and testing for destructive thyroiditis and asymptomatic hypothyroidism

Thyroid hormone levels were measured in 341 subjects in all participants. Thyroid-stimulating hormone (TSH), and free triiodothyronine (FT3), and free thyroxine (FT4) were measured by the CLEIA assay using Lumipulse Presto II (FUJIREBIO Inc., Tokyo). Institutional reference values are as follows: TSH 0.75-4.12 μIU/mL, FT3 2.51-3.47 pg/mL, FT4 0.68-1.26 ng/mL. Anti-thyroglobulin antibody (TgAb) and anti-thyroid peroxidase antibody (TPOAb) levels were measured by ECLIA method using cobas e 801 (Roche Diagnostics K.K, Switzerland). TgAb 40 IU/mL or higher and TPOAb 28 IU/mL or higher were considered positive. Thyroid ultrasonography was performed by at least two skilled sonographers with ultrasonographic equipment, Aplio series (CANON MEDICAL SYSTEMS, Tochigi, Japan).

Destructive thyroiditis was defined as patients who had no abnormal thyroid function before treatment with ICIs, but after the start of treatment, had findings such as hypoechogenicity on thyroid ultrasonography, and who newly needed hormone replacement therapy. Asymptomatic hypothyroidism was defined as cases of elevated TSH or decreased FT3 and FT4 without subjective symptoms of hypothyroidism. Secondary hypothyroidism was defined as a disorder of TSH secretion from the pituitary gland in thyrotropin-releasing hormone (TRH) load test accompanied by decrease of FT3 and FT4.

### Diagnosis and testing for hypoadrenocorticism and hypopituitarism

Adrenocorticotropic hormone (ACTH) and cortisol levels were measured in 331 subjects in all participants. Patients with cortisol deficiency symptoms such as hyponatremia, hypoglycemia, hypotension, and eosinophilia, and who were diagnosed with cortisol insufficiency by rapid ACTH load test, were defined as hypoadrenocorticism. ACTH was measured by ECLIA method using cobas e 801 (Roche Diagnostics K.K, Switzerland), and cortisol was measured by CLIA method using the ADVIA Centaur XP (Siemens Healthcare Corp., Tokyo). In the rapid ACTH load test, 250 μg of tetracosactide was administered intravenously, and blood cortisol was measured before, 30 minutes and 60 minutes after the loading. Sixty minutes later, cortisol levels should be 2-4 times higher than before the loading to diagnose adrenal insufficiency. Since a rapid ACTH stress test may not accurately evaluate acute hypopituitarism especially in a highly stressed state, the diagnosis was made by comprehensively comparing the results with clinical findings such as physical examination and blood tests.

The diagnosis of ACTH isolated deficiency was performed in patients with signs of adrenal insufficiency, ACTH deficiency in corticotropin-releasing hormone (CRH) load test, and no other pituitary hormone deficiency symptoms. In the CRH load test, 100 μg of CRH was administered intravenously after at least 30 minutes of bed rest during early morning fasting, and ACTH was measured at 30, 60, and 90 minutes before the loading. Samples were collected in test tubes containing EDTA, cooled in ice, and centrifuged immediately after the testing. We considered that subjects were ACTH deficient when peak ACTH levels were less than 1.5 times the pre-load level and less than 30 pg/mL.

Panhypopituitarism was defined as cases with multiple endocrine deficiency symptoms associated with low pituitary hormone levels. Diagnostic load testing was performed in 26 cases where it was determined that the malignancy would not affect treatment and admission to the endocrinology department was possible. The CRH load test, growth hormone-releasing peptide-2 (GHRP-2) load test, TRH load test, luteinizing hormone-releasing hormone (LHRH) load test, and saline load test were performed.

### Diagnosis and testing for insulin-dependent diabetes

Endogenous insulin secretory capacity was measured in 110 subjects in all participants with newly detected hyperglycemia or who were attending a diabetes clinic. Insulin-dependent diabetes mellitus was defined as patients who did not require insulin therapy prior to the administration of ICIs, and whose endogenous insulin secretory capacity was assessed by HOMA-β, C-peptide (CPR) index, 24-hour urinary CPR excretion or glucagon tolerance test after the administration of ICIs, and who were newly determined to be in an insulin-dependent state. HOMA-β was calculated by the formula (fasting insulin levels)×360/((fasting plasma glucose levels)-63) and CPR index was calculated by the formula (fasting C-peptide levels)/(fasting plasma glucose levels)×100. We considered that subjects are in insulin-dependent status when metabolic abnormalities such as ketosis and hyperglycemic emergencies were observed. Serum insulin levels were measured by the CLEIA assay using Lumipulse Presto II (FUJIREBIO Inc., Tokyo), and serum CPR levels were measured by ECLIA method using cobas e 801 (Roche Diagnostics K.K, Switzerland).

### Statistical analysis

Data are expressed as mean and standard deviation. The primary endpoint was to assess the incidence of endocrine-related irAEs. The secondary endpoint was to examine factors that could predict the incidence of endocrine-related irAEs. Chi-square tests were used to evaluate the prevalence of all endocrine-related irAEs and the prevalence of destructive thyroiditis, asymptomatic hypothyroidism, primary hypoadrenocorticism, ACTH-only deficiency, panhypopituitarism, insulin-dependent diabetes, respectively. Differences in patient characteristics between with and without endocrine-related irAEs were evaluated using the Mann-Whitney’s U test and the chi-square test. Kaplan-Meier curves were generated, and log-rank tests were used to evaluate survival with and without endocrine-related irAEs. The mean observation period was calculated as the number of days of survival from the date when ICIs were first administered to September 30, 2022. JMP (16.0.1) was used for analysis and EXCEL for Mac (16.58) for tabulation.

## Results

### Prevalence of endocrine-related irAEs after treatment with various ICIs

**Figure 1:**
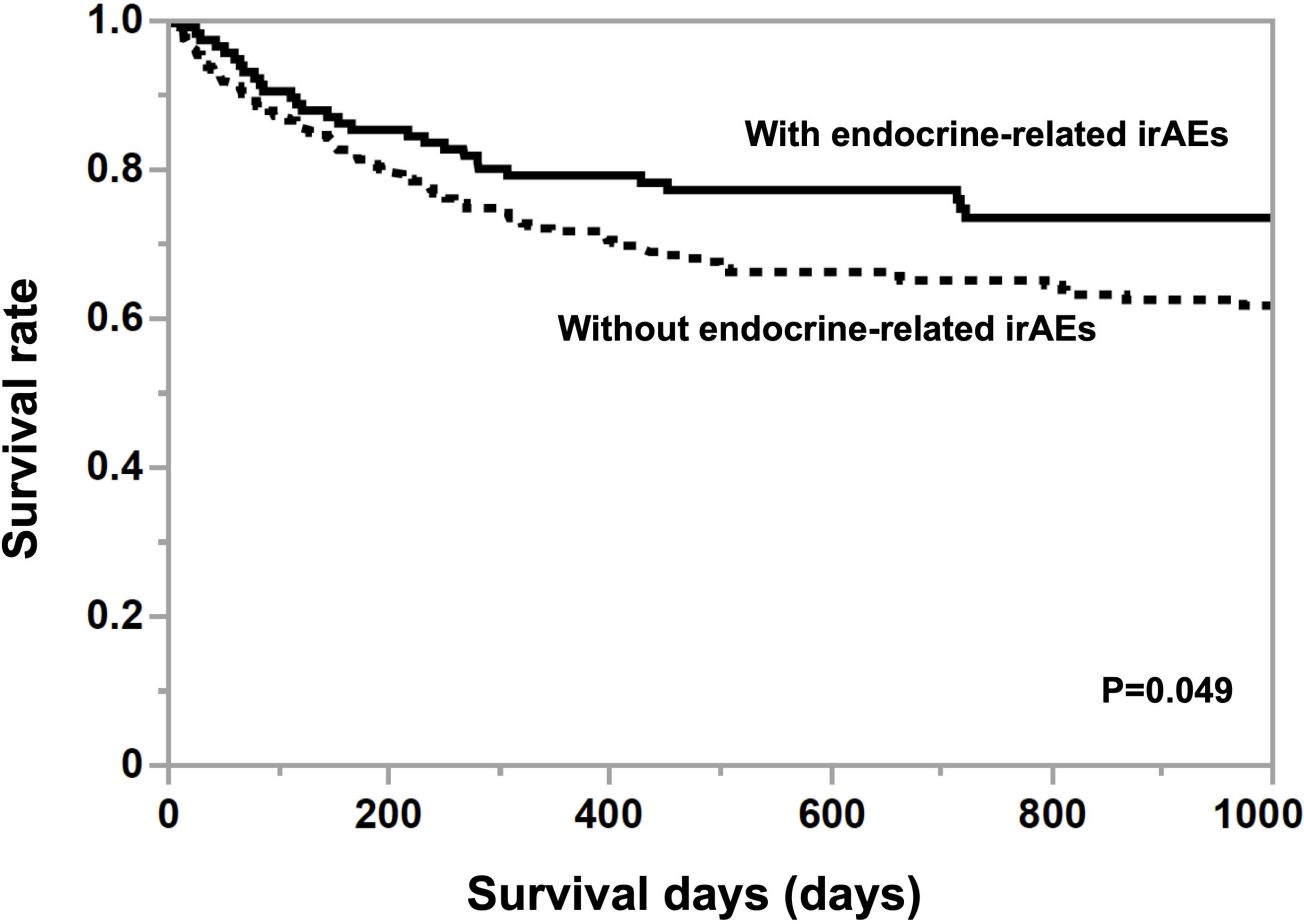
Kaplan-Meier curves in participants grouped by the presence or absence of endocrine-related irAE. A log-rank test was used for analysis, and survival was significantly greater (p<0.05) in participants with endocrine-related irAEs compared to those without endocrine-related irAEs.

The mean age of participants without irAEs was 68.1 ± 1.1 years old and the proportion of women was 25.4% and the mean age of participants with irAEs was 69.1 ± 1.8 years old and the proportion of women was 32.8%. There were no differences in age and gender among the drugs. The mean observation period was 879.8 ± 107.1 days in participants with endocrine-related irAEs, which was significantly longer (p=0.0022) than for those without them (624.2 ± 63.7 days). As shown in **Figure 1**, the Kaplan-Meier curve shows that the 25% life expectancy in participants with and without irAEs was 717 days and 270 days, respectively. Survival rate was significantly higher in participants with irAEs (p=0.049). **Table 1** shows the prevalence of irAEs by primary disease, days to death, and ICIs administered. The prevalence of endocrine-related irAEs was higher in participants with renal cell carcinoma, malignant pleural mesothelioma, and hepatocellular carcinoma (p=0.040). The median number of days in the participants who died during the observation period was 153.5 days. Malignant melanoma and otorhinolaryngological tumors were treated only with PD-1 inhibitors and ipilimumab. Gastrointestinal cancers, hematologic tumors, cancers of unknown primary, and female organ cancers were treated with PD-1 inhibitors. Hepatocellular carcinoma, small cell lung cancer, and breast cancer were treated with atezolizumab.

**Table 1:** Prevalence of endocrine-related immune-related adverse events in subjects with various types of cancer.

	Non-small cell lung cancer (n=158)	Gastrointestinal cancer (n=103)	Hepatocellular carcinoma (n=37)	Otorhinolaryngo-logical cancer (n=35)	Urinary tract cancer (n=34)	Malignant melanoma (n=31)	Renal cell carcinoma (n=28)
Presence of endocrine-related irAEs (%)	22.8	17.5	46.0	6.7	17.7	35.5	53.6
Mortality within 12 months of ICI administration (%)	39.3	34.6	18.8	21.2	31.3	16.1	20.0
Mean time to death (days)	328.1±82.1	202.2±109.0	208.7±257.0	82.4±238.0	192.4±199.9	360.0±281.5	281.5
Immune checkpoint inhibitors administered							
Ipilimumab (%)	3.2	0	0	2.9	2.9	29.0	28.6
Nivolumab (%)	24.7	90.3	0	60.0	0	35.5	35.7
Pembrolizumab (%)	50.6	9.7	0	37.1	91.2	35.5	28.6
Atezolizumab (%)	13.3	0	100.0	0	0	0	0
Dulbumumab (%)	8.2	0	0	0	0	0	0
Avelumab (%)	0	0	0	0	5.9	0	7.1
	Small cell lung carcinoma (n=12)	Hematologic tumor (n=8)	Cancer of unknown primary (n=8)	Malignant pleural mesothelioma (n=4)	Cancer of female genital organs (n=4)	Breast cancer (n=3)	Pancreatic cancer (n=1)
Presence of endocrine-related irAEs (%)	16.7	12.5	12.5	50.0	25.0	0	100
Mortality within 12 months of ICI administration (%)	54.6	12.5	57.1	66.7	33.3	66.7	0
Mean time to death (days)	240.8±257.0	240.0	101.0±314.8	185.5±355.1	40	132.5±445.1	–
Immune checkpoint inhibitors administered							
Ipilimumab (%)	0	0	0	25.0	0	0	0
Nivolumab (%)	0	50.0	62.5	75.0	50.0	0	100
Pembrolizumab (%)	0	50.0	37.5	0	50.0	0	0
Atezolizumab (%)	100	0	0	0	0	100	0
Dulbumumab (%)	0	0	0	0	0	0	0
Avelumab (%)	0	0	0	0	0	0	0

Data are expressed as mean and standard deviation. irAE, immune-related adverse events; ICI, immune checkpoint inhibitor.

### Prevalence of endocrine-related irAEs after treatment with various ICIs

**Table 2** shows the prevalence of irAEs after combination therapy with CTLA-4 and PD-1 inhibitors and monotherapy with PD-1 inhibitors or PD-L1 inhibitors. The overall incidence of any endocrine-related irAEs among the participants was 25.5%. Any hypothyroidism was prevalent in 24.3%, any hypoadrenocorticism in 3.2%, hypopituitarism in 0.9%, and insulin-dependent diabetes mellitus in 1.1% in all participants. Of the 341 participants whose thyroid hormones were measured, any hypothyroidism was present in 103 participants (30.2%). Any hypoadrenocorticism was found in 15 of the 331 participants (4.5%) whose adrenocortical hormones were measured. Insulin-dependent diabetes mellitus was found in 5 of the 110 participants (4.6%) whose insulin secretion capacity was measured. The participants receiving the combination therapy with CTLA-4 and PD-1 inhibitors had a significantly high incidence of irAEs. Destructive thyroiditis, ACTH isolated deficiency, and hypopituitarism were significantly more prevalent in the combination therapy group (p=0.019, p=0.0003, and p=0.0003, respectively).

**Table 2:** Prevalence of endocrine-related immune-related adverse events after treatment with various immune check point inhibitors.

	All participants (n=466)	Ipilimumab and PD-1 inhibitors (n=25)	PD-1 inhibitors (n=351)	PD-L1 inhibitors (n=90)
Endocrine-related irAEs (%)	25.5	52.0	23.1	27.8
Any hypothyroidism (%)	24.3	52.0	21.7	26.7
Destructive thyroiditis (%)	9.4	24.0	6.8	15.6
Asymptomatic hypothyroidism (%)	14.2	24.0	14.3	11.1
Secondary hypothyroidism (%)	0.4	0	0.6	0
Any hypoadenocorticism (%)	3.2	20.0	1.7	4.4
Primary hypoadrenocorticism (%)	0.6	0	0.6	1.1
ACTH isolated deficiency (%)	2.2	12.0	1.4	2.2
Hypopituitarism (%)	0.9	12.0	0	1.1
Insulin-dependent diabetes (%)	1.1	0	1.4	0

irAE, immune-related adverse events; PD-1, programmed cell death protein 1; PD-L1, programmed cell death protein 1 ligand 1; ACTH, adrenocorticotropic hormone.

### Difference in the prevalence of endocrine-related irAEs among various ICIs

The prevalence of irAEs by drug is shown in **Table 3**. The prevalence of any endocrine-related irAEs was more frequent in participants treated with the combination of ipilimumab and PD-1 inhibitors, followed by avelumab, atezolizumab, nivolumab, and pembrolizumab (p=0.017). The prevalence of hypothyroidism, hypoadrenocorticism, and hypopituitarism was higher among the participants who received ipilimumab plus a PD-1 inhibitor; among participants who received ICI alone, the prevalence of any type of hypothyroidism was higher in participants treated with atezolizumab, nivolumab, and pembrolizumab in this order. Atezolizumab also had a higher prevalence of primary hypoadrenocorticism and insulin-dependent diabetes mellitus than other ICIs.

**Table 3:** Prevalence of endocrine-related immune-related adverse events after treatment with various immune checkpoint inhibitors.

	Ipilimumab and PD-1 inhibitors (n=25)	Nivolumab (n=189)	Pembrolizumab (n=162)	Atezolizumab (n=73)	Dulbumumab (n=13)	Avelumab (n=4)
Endocrine-related irAEs (%)	52.0	23.3	22.8	30.1	7.7	50.0
Any hypothyroidism (%)	52.0	22.8	20.4	28.8	7.7	50.0
Destructive thyroiditis (%)	24.0	5.3	8.6	16.4	7.7	25.0
Asymptomatic hypothyroidism (%)	24.0	17.5	10.5	12.3	0	0
Secondary hypothyroidism (%)	0	0	1.23	0	0	0
Any hypoadenocorticism (%)	20.0	0.5	3.1	4.1	0	25.0
Primary hypoadrenocorticism (%)	0	0	1.2	1.4	0	0
ACTH isolated deficiency (%)	12.0	0.5	2.5	2.7	0	0
Hypopituitarism (%)	12.0	0	0	0	0	25.0
Insulin-dependent diabetes (%)	0	1.1	1.9	10	0	0

irAE, immune-related adverse events; PD-1, programmed cell death protein 1; PD-L1, programmed cell death protein 1 ligand 1; ACTH, adrenocorticotropic hormone.

### Thyroid antibodies and destructive thyroiditis

**Figure 2:**
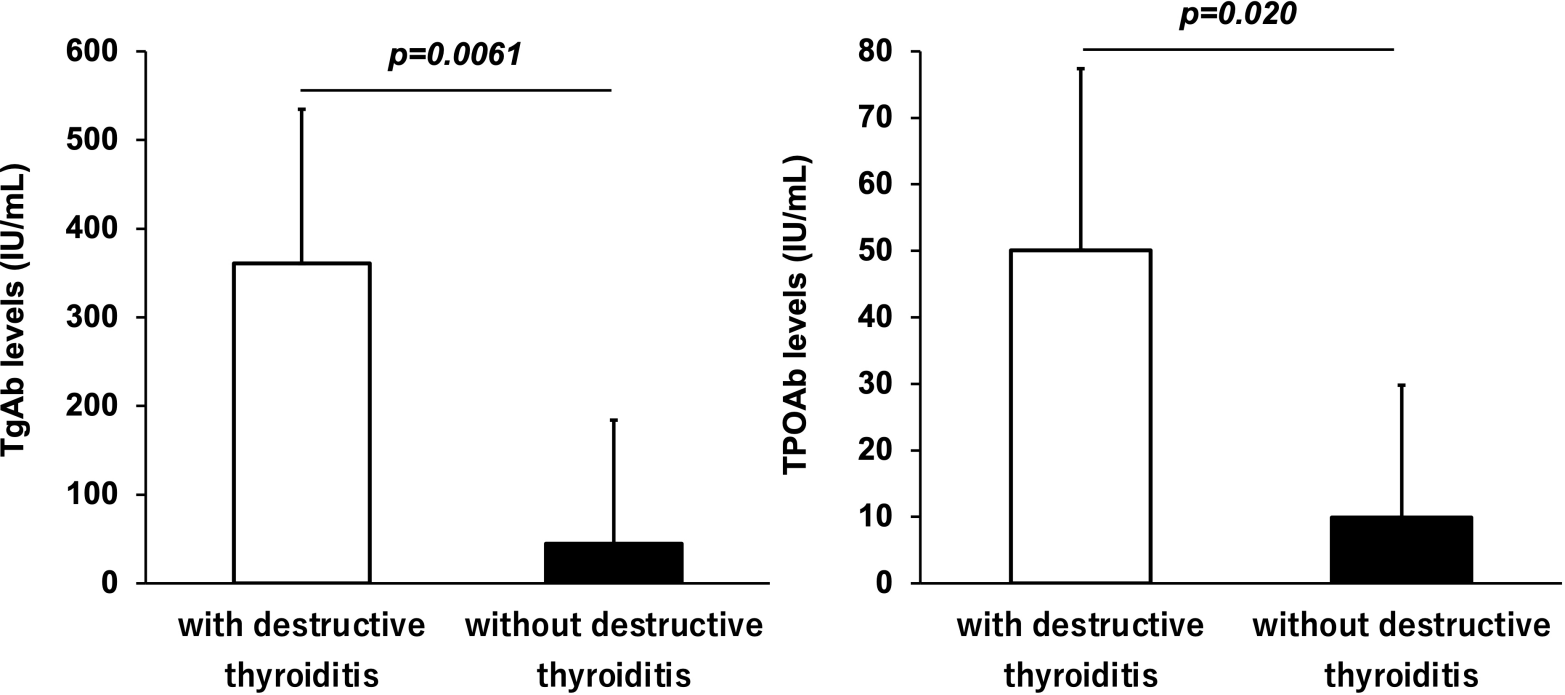
Antithyroglobulin antibody (TgAb) and antithyroid peroxidase antibody (TPOAb) levels in subjects with and without destructive thyroiditis. TgAb and TPOAb levels were significantly higher in participants who developed destructive thyroiditis than those without it (p < 0.05, respectively).

Among the participants who received ICIs, TgAb and TPOAb were measured in 69 and 77 participants, respectively. **Figure 2** shows the antibody levels of TgAb and TPOAb in participants with and without destructive thyroiditis. The mean TgAb level in the participants who developed destructive thyroiditis was 360.8 ± 173.9 IU/mL, which was significantly higher (p=0.0061) than the mean TgAb level of 44.7 ± 139.4 IU/mL in those without it. Destructive thyroiditis occurred in 13 (76.5%) of 17 TgAb-positive patients. The incidence of destructive thyroiditis was significantly higher in the TgAb-positive participants than in the negative participants (p=0.024). Similarly, participants who developed destructive thyroiditis had a mean TPOAb of 50.1 ± 27.3 IU/mL, which was significantly higher than the mean TPOAb of 9.9 ± 19.9 IU/mL in those without it (p=0.020).

### Causes of hypoadrenocorticism

Fifteen of 466 participants developed hypoadrenocorticism after treatment with ICIs. The causative diseases were ACTH isolated deficiency in 10 participants, primary hypoadrenocorticism in 2 participants, a combination of ACTH isolated deficiency and primary hypoadrenocorticism in 1 participant, and hypoadrenocorticism associated with panhypopituitarism in 2 participants. The mean time to development of hypoadrenocorticism after ICI administration was 213.3 ± 159.5 days; the mean (min-max) time to development of ACTH isolated deficiency was 219.3 ± 106.1 (13-591) days. The time to onset of primary hypopituitarism and hypopituitarism associated with ACTH isolated deficiency and panhypopituitarism were 370, 64, and 308 days, respectively.

### Clinical background of insulin-dependent diabetes mellitus

All five participants who were diagnosed with insulin-dependent diabetes mellitus after treatment with ICIs were treated with PD-1 inhibitors. Only 1 of the 5 participants was positive for anti-glutamic acid decarboxylase antibody (GADAb). Three of the 5 participants were diagnosed with type 2 diabetes prior to treatment with ICIs and were treated with oral hypoglycemic agents. The mean time from administration of ICIs to insulin therapy was 177.4 ± 98.3 days.

## Discussion

These retrospective data revealed the incidence of endocrine-related irAEs in participants who received ICIs. We also evaluated differences in the incidence of endocrine-related irAEs among various ICIs and differences in survival between with and without irAEs. To date, there have been no studies of endocrine-related irAEs exclusively in Japanese subjects, and the data from this study clarify the characteristics of endocrine-related irAEs caused by the administration of ICIs.

Endocrine-related irAEs such as hypoadrenocorticism and insulin-dependent diabetes mellitus are a challenge to diagnose and treat appropriately because the hormone deficiency symptoms can lead to decreased ADL and death (2). Studies in non-small cell lung cancer and other tumors have found a significant correlation between the development of irAEs and survival (3, 4). Studies in head and neck and lung cancer have reported that the development of endocrine-related irAEs significantly improved patient survival (5). In this study, the presence of endocrine-related irAEs diagnosed by blood tests was significantly correlated with survival. While appropriate treatment with ICIs can be expected to improve survival in patients with malignant tumors, it also indicates the need to be vigilant for the development of irAEs (6).

It has been reported that the incidence of endocrine-related irAEs is increased when multiple immune checkpoint inhibitors are used compared to monotherapy (7) and that the incidence of hypothyroidism with immune checkpoint inhibitors is higher with CTLA-4 inhibitors, PD-L1 inhibitors, nivolumab, pembrolizumab, and CTLA/4/PD-1 inhibitor combination therapy, in this order. The incidence of endocrine-related irAEs in patients treated with ipilimumab plus PD-1 inhibitors in this study was significantly higher (52.0%), with hypothyroidism accounting for the majority. The incidence of ACTH isolated deficiency and hypopituitarism was 12% each, both of which were significantly higher compared to monotherapy. The incidence of ACTH isolated deficiency and hypopituitarism was also higher than that of monotherapy at 12% each. However, the incidence of endocrine-related irAEs with combination therapy was higher than previously reported.

TgAb/TPOAb positivity is associated with the development of hypothyroidism after administration of ICIs (8). In this study, 76.5% of TgAb-positive patients developed destructive thyroiditis. TgAb and TPOAb were significantly higher in patients who developed destructive thyroiditis than in those without it. Measurement of these antibodies prior to administration of ICIs may be an important marker for predicting the development of destructive thyroiditis.

A meta-analysis in hypoadrenocorticism as irAEs showed a frequency of 5.3% for primary hypoadrenocorticism, 92.7% for secondary hypoadrenocorticism, and 1.9% for mixed type (9). In the present study, among 466 cases, 1 was primary hypoadrenocorticism, 11 were secondary hypoadrenocorticism, and 1 was mixed hypoadrenocorticism. The mechanism by which ICIs induce ACTH isolated deficiency and the mechanism of action of PD-1 inhibitors on the pituitary gland are not clear. Previous studies have shown that ACTH isolated deficiency can be caused by both CTLA-4 and PD-1/PD-L1 inhibitors (10).

The reported frequency of insulin-dependent diabetes after treatment with ICIs is 0.84% (11). In this study, 1.1% of all participants were newly diagnosed with insulin-dependent diabetes mellitus after receiving ICIs, which was similar to the previous report. It has been reported that patients with a history of type 2 diabetes are more likely to develop insulin-dependent diabetes (12). In this study, 3 of the 5 patients who were diagnosed with insulin-dependent diabetes had a history of type 2 diabetes. The prevalence of anti-GAD antibodies was 51% (13), but only 1 among the 5 patients in this study. Insulin-dependent diabetes mellitus as irAEs is an infrequent condition, and further studies with a larger number of patients are needed.

There are several limitations in this study. First, this study is a single-center, retrospective, observational study. All participants in this study are Japanese, and the results of this study may not reflect the situation in other countries due to racial differences or differences in treatment strategies from other countries. Second, endocrine testing was not performed in all patients. Hypoadrenocorticism and insulin-dependent diabetes mellitus can be detected in most cases because of the appearance of serious clinical signs but may not be detected in cases of mild disorders. Next, survival and mean observation time were longer in participants with irAEs compared to those without irAE. These data suggest that more endocrine-related irAEs are brough about in participants who survived for a longer period. The response rate, dosage of ICIs and the method of chemotherapy were not studied in this study. Finally, there was variation among the number of cases for each drug; compared to PD-1 inhibitors and atezolizumab, smaller numbers of cases were treated with durvamumab and avelumab. Further study is needed to determine the effect of ipilimumab monotherapy, as no participants received it as monotherapy.

In conclusion, endocrine-related irAEs correlated significantly with survival and mean observation period. The clinical aspects of participants who developed each endocrine-related irAEs were partially clarified, and the development of endocrine-related irAEs should be noted in situations where treatment with ICIs can be expected. The incidence of endocrine-related irAEs due to ICIs was higher in the study subjects than previously reported, and endocrine testing should be performed regularly in patients treated with ICIs to watch for endocrine-related irAEs.

## Data availability statement

The original contributions presented in the study are included in the article/supplementary material. Further inquiries can be directed to the corresponding author.

## Ethics statement

The studies involving human participants were reviewed and approved by Institutional Review Board of Kawasaki Medical School. Written informed consent for participation was not required for this study in accordance with the national legislation and the institutional requirements.

## Author contributions

YI designed the study. YI, TK, HI, JS, YF, YK, FT, MS, SN, TM, and HK treated patients and collected data. YI analyzed the data. YI, TK, FT, MS, SN, TM and HK contributed to discussion. KK supervised the project. YI wrote the manuscript. HK reviewed and edited the manuscript. All authors contributed to the article and approved the submitted version.
